# Patriotism and the Nurse-Training Schools

**Published:** 1915-12-04

**Authors:** 


### PATRIOTISM & THE NURSE-TRAINING SCHOOLS.*

### V. SOME PROVINCIAL POOR-LAW INFIRMARIES.

The services rendered to the sick and wounded in provincial infirmaries are not to be measured by the numbers who have gone abroad from the nursing staff or taken up war work in military hospitals in various parts of England. In every Union infirmary where the accommodation was considered suitable, arrangements have been made to set apart a large proportion of the beds for the use of the War Office. Many Poor-Law infirmaries all over the country have been turned into military establishments, and have undergone strange transformation in the process. Under such circumstances the duty of the staff was plainly to remain at their posts, and the losses have chiefly been experienced in the nursing departments of those institutions not taken over for the soldiers till the spring of this year. We give some examples of the manner in which modifications have taken place in these establishments.

### Crumpsall Infirmary, Manchester.

This famous training school for nurses has now become a branch of the 2nd Western Military Hospital in Manchester. Since the end of October 1914, when 250 cases were admitted in three days, the energies of the nursing staff have been devoted to the care of the wounded, first Belgian and later on British soldiers. There are 500 beds reserved for the disposal of the War Office, but so far not more than 365 have been occupied at the same time. The nursing has been carried out entirely by the infirmary staff, the greatest possible test of an efficient training school, and only one member of it, Miss Ada Hopwood, now in charge of the Filey Street Military Hospital in one of the Council schools taken over by the War Office, is on war service outside. We understand that her post will be kept open for her. A new $x$-ray department was opened in the infirmary last year, and has proved invaluable since the arrival of the wounded.

### Shirley Warren Infirmary, Southampton.

An auxiliary hospital of seventy-four beds—one of the new blocks in the grounds of the institution —has been devoted to wounded soldiers, who are being nursed by the infirmary staff. Of this, Sister Widger is Sister-in-Charge. One of the sisters, Miss Mary Hayes, volunteered at the outset of the war for foreign service, and was accepted by the St. John Ambulance Association. She left at once for Antwerp, and was there during the whole of the bombardment. After returning to London she was appointed to the Sophie Berthelot Hospital in Calais. Nurse Frances Butler has been appointed to the Alexandra Hospital, Cosham. Nurses M. Pickslock, Dixon, A. Matthews, G. Parker, and M. Ford are serving in military hospitals at home. Two former members of the staff, Nurses Pottinger and E. M. Lyle, have been sent to Malta and France respectively. We understand that several sisters besides those named were anxious to volunteer for war service, but seeing that they were badly wanted in their own institution owing to the heavy increase of work and the shortness of the staff, they decided to remain. All honour to them. They have done their duty well.

### Township Infirmary, Leeds.

In July, 1914, Miss Fanny Spann was appointed matron of this infirmary. Being a member of the Territorial Force Nursing Service she was called up within a month for active service. Upon the urgent request of the Guardians, however, she resigned her position in the Territorial Service and in September took up duty in the infirmary, where she has remained ever since. The assistant matron, Miss E. Storey, was at once set free for active service, together with Sisters Wood and Wright (T.F.N.S.), and Sisters Kennedy, Bebb, and Wilson (military hospitals at home). The following staff-nurses also left:—Nurses Lee, Stafford, Hare, Wilkinson, Verity, Aske, Wharton, Westridge, (T.F.N.S.), Nurses Harris and Fletcher (in Malta), and Nurses O'Donnell and Spindler (Q.A.I.M.N.S.R.) A large number of nurses trained in the institution are doing hospital work in connection with the war. Six V.A.D. members have been received each month during the past year for preparatory ward work. Several sick Belgians have been nursed in the infirmary, but the wounded soldiers are being nursed in the workhouse blocks which the Guardians have temporarily transferred to the military authorities, and these are staffed by Territorial nurses under Territorial authority.

### Stapleton Institution Infirmary, Bristol.

Nurses H. E. Mason, E. A. Speary, and E. Smith left the institution on July 25, 1915, for the Dardanelles. Nurses G. Senington, A. Montagu, and D. Oakhill have taken up war duty in home hospitals.

### Stockport Union Infirmary.

This institution is now converted into an auxiliary military hospital, and furnishes 340 beds for wounded soldiers. In addition to the members of the staff doing war work in their own establishment, Sister K. O'Boyle is working in Alexandria (Q.A.I.M.N.S.), and Sisters Booth and Evans have joined the 2nd Western Military Hospital.

### Merthyr Tydvil Union.

Charge Nurse Elvina Davies is at Lord Derby's War Hospital, Warrington; and Nurse Letitia Rees is nursing the wounded at the 1st Southern Military Hospital, Birmingham.

### Cardiff Union Hospital.

Sisters Letty Cash, Lucy Vickery, and O. Phillips have resigned from the staff and have joined the Territorial Force Nursing Service. They are working at the 3rd Western Territorial Hospital.

* Previous articles appeared on October 23, October 30, November 6, and November 20.

